# The interplay between maze complexity, colony size, learning and memory in ants while solving a maze: A test at the colony level

**DOI:** 10.1371/journal.pone.0183753

**Published:** 2017-08-24

**Authors:** Maya Saar, Tomer Gilad, Tal Kilon-Kallner, Adar Rosenfeld, Aziz Subach, Inon Scharf

**Affiliations:** School of Zoology, Faculty of Life Sciences, Tel Aviv University, Tel Aviv, Israel; University of California San Diego, UNITED STATES

## Abstract

Central-place foragers need to explore their immediate habitat in order to reach food. We let colonies of the individually foraging desert ant *Cataglyphis niger* search for a food reward in a maze. We did so for three tests per day over two successive days and an additional test after a time interval of 4–20 days (seven tests in total). We examined whether the colonies reached the food reward faster, consumed more food and changed the number of workers searching over time, within and between days. Colonies’ food-discovery time shortened within and between days, indicating that some workers learnt and became more efficient in moving through the maze. Such workers, however, also forgot and deteriorated in their food-discovery time, leveling off back to initial performance after about two weeks. We used mazes of increasing complexity levels, differing in the potential number of wrong turns. The number of workers searching increased with colony size. Food-discovery time also increased with colony size in complex mazes but not in simple ones, perhaps due to the more frequent interactions among workers in large colonies having to move through narrow routes. Finally, the motivation to solve the maze was probably not only the food reward, because food consumption did not change over time.

## Introduction

Learning can be defined as the acquisition of experience, allowing an animal to change its response to specific stimuli or situations [1–2]. There are numerous studies showing that insects can learn to associate a cue with a reward, as either classical conditioning or operant learning (reviewed in: [1, 3]). Learning is considered to have evolved due to its positive contribution to fitness, although there has been little study to support this (but see, e.g., [4–5]). Most animals undergo some learning process while foraging, from movement strategies, through the value of specific prey types, to the best way of subduing the prey [6–8]. Learning abilities in the search for food resources should evolve, especially when resources are clumped in space or only temporarily available [9]. Central place foragers, such as ants and rodents living in nests, need to navigate in space not only in order to locate food but also to bring it back to their nest, in a way that minimizes travel and time costs [10–12]. This requirement has selected for enhanced spatial orientation in such animals [13–14].

Under laboratory settings, mazes are a common way to test spatial orientation, using different animals, from insects to humans [15–17]. Maze solving in insects has been previously tested mostly in cockroaches and ants ([18–21]; but also *Drosophila*; [15]). Studies on ants disentangled between the contribution of different cues for learning, such as visual and chemical cues [22–23]. More recent studies showed that ants learnt better repeating routes than alternating ones (e.g., successive right turns; [24]), and that ants can associate right-left turns with landmarks of different widths [25]. Experiments on maze solving in ants have used group-foraging ants, such as *Solenopsis*, *Formica* or *Lasius* species [18, 20, 22, 24, 26], while individually foraging ants have been studied to a lesser extent (but see [25, 27]). Furthermore, colony size has been rarely taken into account (but see, e.g., [26]), and the complexity level of the experimental set-up (e.g., maze) has usually been ignored (but see [28–29]).

Memory can be defined as the ability to store and retrieve information from the past [30]. Memory is constrained by various costs, such as maintaining the brain (a cost an animal pays whether it learns or not), a trade-off between energy devoted to memory and other needs, and time and energy invested in the learning process [31]. The duration of memory or how fast information is forgotten is an important but less often studied trait, exposed to natural selection [32–33]. Good examples of adaptive forgetting is when the environment changes, such as location of objects in the habitat used for navigation, when learning a new task interferes with the memory of an older one, or when the learning of an old food location slows down learning of a new food location [34–37]. Generally, there should be a negative correlation between the rate of environmental change and memory retention [14].

Social insects display two levels of organization: the individual level and the colony level. Because fitness of social insects is determined at the colony level, different collective behaviors, such as colony defense, nest construction/relocation and foraging, greatly impact survival and reproduction; many behavioral studies, therefore, focus on that level [38–40]. We studied here the contribution of learning within and between days to maze solving in a desert individually foraging ant (with no recruitment under foraging context), *Cataglyphis niger*. We combined three often-ignored aspects of learning in ants: (1) different complexity levels of the maze; (2) colony size, and (3) memory duration. Learning to solve a maze at the individual level translates into an earlier arrival to the food reward and its more intense exploitation. The colony, however, can also flood the maze with foragers, which would lead to a shorter maze-solving time over successive tests, with little requirement for individual learning. A good example of improvement at the colony level with experience is the nest relocation time demonstrated by cavity-dwelling ants [41].

We had four main goals: (1) to examine the effect of maze complexity and colony size on food-discovery time, number of workers searching and food consumption; (2) to examine whether colonies reach the food reward faster with experience in within- and between-day comparisons; (3) to test whether the number of workers searching and food consumption increase with experience as well; and (4) to study whether ants forget and deteriorate in solving the maze after a longer time interval. We expected: (1) a positive effect of colony size on the number of workers searching, food consumption and food-discovery time, while high maze complexity should have the opposite effect; (2) a decline in food-discovery time with experience when tested on the same day or between two successive days; (3) an increase in the number of workers searching and food consumption with experience; and (4) a forgetting process of the maze and hence an increase in food-discovery time over long time intervals.

## Methods

### General procedure

**Fig 1:**
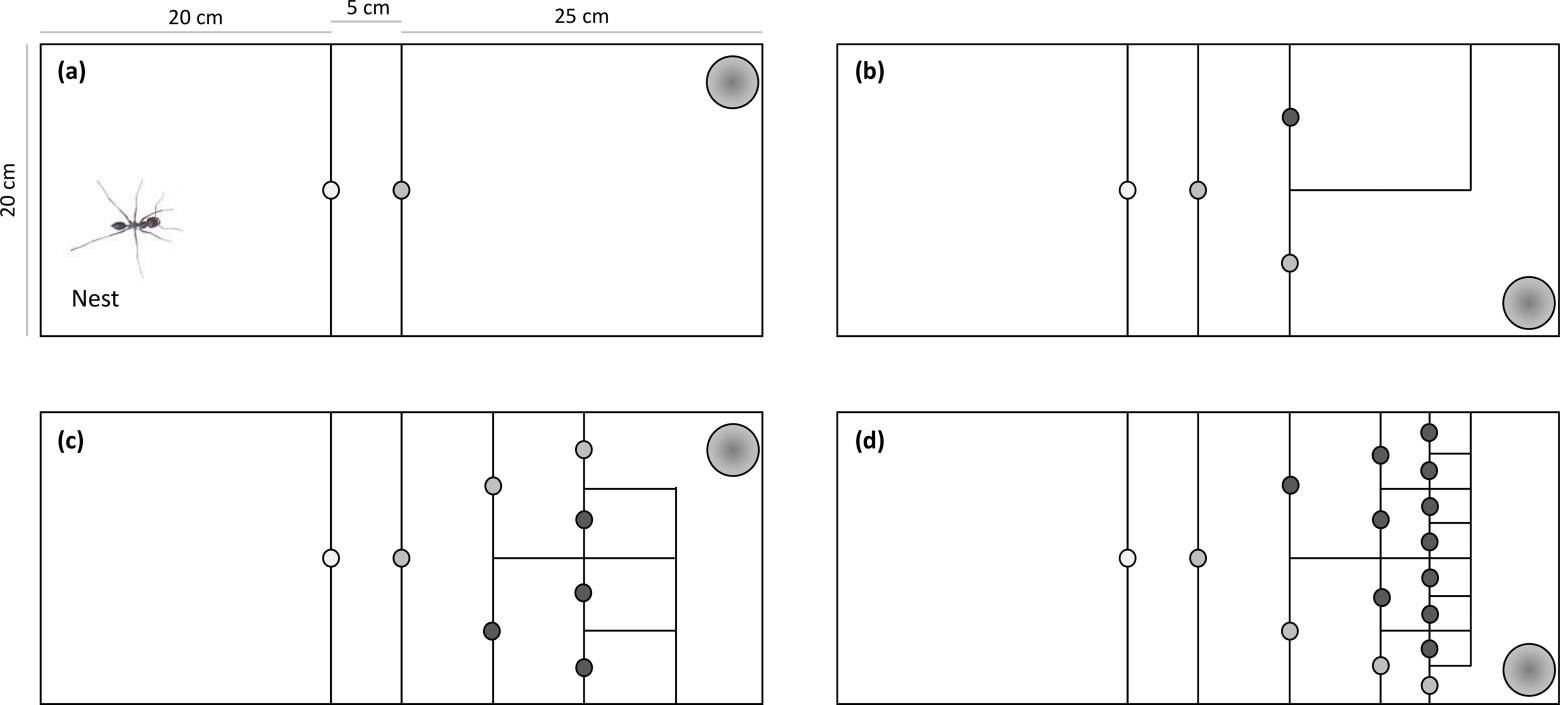
Illustration of a nest and a maze of complexity levels (a) 0, (b) 1, (c) 2 and (d) 3. Each circle represents a passage and a decision to make (light gray ones are correct decision, leading to the food reward, and dark grey ones are wrong decisions). The larger circle on the right upper (a, c) side or right lower side (b, d) represents the food reward (honey). The correct number of decisions, including the decision to enter the maze are 1, 2, 3, and 4 for complexity levels 0 to 3, respectively. The total number of decisions are 1, 3, 7, and 15 for the same complexity levels. Mazes included either right or left turns as the correct ones, which were randomly applied per colony.

26 complete queenright colonies (N _workers_ = 146.6 ± 58.5; mean ± 1 SD; range [45,265]) were excavated in the Tel Baruch sand dunes (32.1283N, 34.7867E; ~20 m above sea level) during May-December 2016. Colonies were moved to laboratory conditions (~28°C, ~50% relative humidity) and kept under dark conditions. They were fed upon arrival with 0.5 g of honey (from a single source) in 6-cm petri dishes, cut in the margins to allow easy access. Five hours later the honey plates were removed for a starvation period of 7–10 days before the experiment started. The colonies remained starved until the end of the experiment. We preferred to starve the colonies throughout the experiment and not to feed them ad-libitum first, in order to keep both foraging intensity and motivation to learn high during all steps of the experiment. *C*. *niger* colonies can resist starvation for months (at least four months under laboratory conditions; pers. observ.), so the starvation period applied here is not expected to induce harsh starvation. Before the experiment began we photographed the colony to determine its colony size, but a manual count was also done in most cases and compared to the photography count. The colonies were kept in Perspex cages (50×20×5 cm; l×w×h). Each cage was divided into three sections, connected with detachable doors (Fig 1): (1) the nest (20×20 cm) containing three 8-cm glass tubes filled with water and sealed with cotton wool to increase humidity level, (2) a control strip (5×20 cm), and (3) an open arena in which to insert the test maze (25×20 cm; see below). Each colony was randomly assigned with a maze of four different complexity levels (0–3). The maze was composed of a sequence of binary choices of correct vs. wrong turns. The number of possible wrong decisions while searching increases with the complexity level. The number of correct decisions is equal to the complexity level (plus one, if we consider the decision to enter the maze). Mazes of higher complexity are more difficult to solve, because the number of wrong decisions increases faster than the number of correct decisions (Fig 1; see also ‘Supporting Information‘ for a more thorough description of the maze). The mazes included either correct right turns or correct left turns, which were randomly applied for different colonies. It is likely that a maze with a mixture of correct turns (e.g., right-left-right) would challenge the ants more than a maze with either a constant right or left turn [24]. Still, learning that the correct turn is always right or left and applying this rule still requires more trial-and-error, effort and time than just turning right or left once. In this sense, it fulfills the goal of this experiment: testing the ants in mazes, which an increasing amount of time is needed to solve them.

At the opposite end of the maze we inserted a 6-cm petri dish with 0.5 g of honey (Fig 1). The experiment began while all the ants were in the nest and the detachable doors were all removed at once, enabling free passage to the control strip and then to the maze arena. During each test, we measured three foraging behavioral response variables: (1) Food-discovery time: the time required for the first worker to enter the correct cell of the maze and then arrive at the honey plate. (2) Workers searching: the number of workers present in the test maze and the control strip, exactly when the first worker solved the maze (i.e., entered the correct cell of the maze). (3) Food consumption: we weighed the honey before and after each test (accuracy of 0.1 mg) to calculate how much was consumed during the 10 minutes that the workers were allowed to feed. This is also the duration of each test: time to arrive at the honey plate (a median of 280 seconds with a range of 14–2232 seconds) plus 10 minutes.

Each colony (n = 26) was tested three times on the first day; these colonies were included in the two first analyses (within-day analyses; see below). Nineteen of the colonies were tested at least once more on a successive day and were used in the between-day analysis (the third analysis; see below). Of those 19 colonies, 18 colonies were tested once more after a longer interval of 11.1 ± 5.6 days (mean ± 1 SD; range: [4, 20]). Colonies were randomly assigned to intervals, with no correlation of the interval either with colony size or with complexity level (P = 0.591 and 0.872, respectively). The decision whether to include a colony or not in a specific analysis depended only on the availability of data. Between successive tests within a single day, there was an interval of 30 min in which ants were returned to their nest, and the maze arena was cleared with alcohol to avoid residual odors. The three foraging response variables were log_10_-transformed due to their right-skewed distribution. There were no required permits for the collection of *C*. *niger*, as we collected this common, unprotected species in the municipal area of Tel Aviv. All colonies survived the experiments, which were not harmful, and colonies were further kept in the laboratory for another, longer-term research. All applicable guidelines for the care and use of animals were followed.

### Data analyses

#### The effect of maze complexity and colony size on foraging response variables

We examined whether there is an effect of maze complexity (0–3) and colony size (number of workers) on the three foraging response variables (food-discovery time, workers searching, and food consumption). We focused only on the first test on the first day and each colony was considered only once to avoid pseudo-replications. We used three separate linear regressions, with colony size, maze complexity and their interaction as explanatory variables. We did not remove non-significant interactions, and present full models.

#### Within-day changes in foraging response variables

To test for within-day changes we calculated for each colony the linear regression slope over the three tests of day 1 for each of the three foraging response variables (each colony was used only once). We performed two analyses: (1) One-sample t-tests to determine whether the slopes of each behavioral variable come from a distribution with a mean of zero, indicating no change with successive tests (H0). If the confidence intervals of the slopes do not overlap zero, we can determine that there had been significant changes (increase or decrease) within days. (2) To determine whether within-day changes were affected by colony size and maze complexity, we tested the effects of these two factors on the slopes of the three foraging variables using linear regressions. Full models are presented.

#### Changes between successive days in foraging response variables

We compared the first test on the first and second days of testing for each of the three foraging response variables using repeated-measures ANCOVA, with day as the within-subject factor, and colony size and complexity level as the between-subject factors. We chose the first test of both days, because on day 1 it represents the initial level of foraging, prior to any potential learning procedure, and on day 2 it represents the first test on that day, after a 24 h interval. If there is a learning process between days, food-discovery time should be lower on the second day. Full models are presented.

#### Foraging response variables over longer time intervals

We compared the values of each of the three foraging response variables on the first day and test of the experiment and on the first test after the longer interval of 4–20 days using repeated-measures ANCOVAs with day as the within-subject factor and the time interval as the between-subject factor. Colony size and complexity were not included here, because their effect was already tested before.

## Results

### The effect of maze complexity and colony size on foraging response variables

Statistical results are summarized in Table 1.

**Table 1:** The effect of colony size and maze complexity on the three foraging response variables on the first day and test.

Foraging variable	Colony size	Maze complexity	Interaction term	Model
Food-discovery time	t = -0.785, P = 0.441	**t = -2.779,** **P = 0.011**	**t = 2.945,** **P = 0.007**	N = 26, R_2_ = 0.308
Workers searching	t = 1.075, P = 0.294	t = -0.564, P = 0.579	t = 1.023, P = 0.317	N = 26, R_2_ = 0.292
Food consumption	t = 0.248, P = 0.807	t = 1.615, P = 0.123	t = -1.467, P = 0.159	N = 23, R_2_ = 0.128

#### Food-discovery time

**Fig 2:**
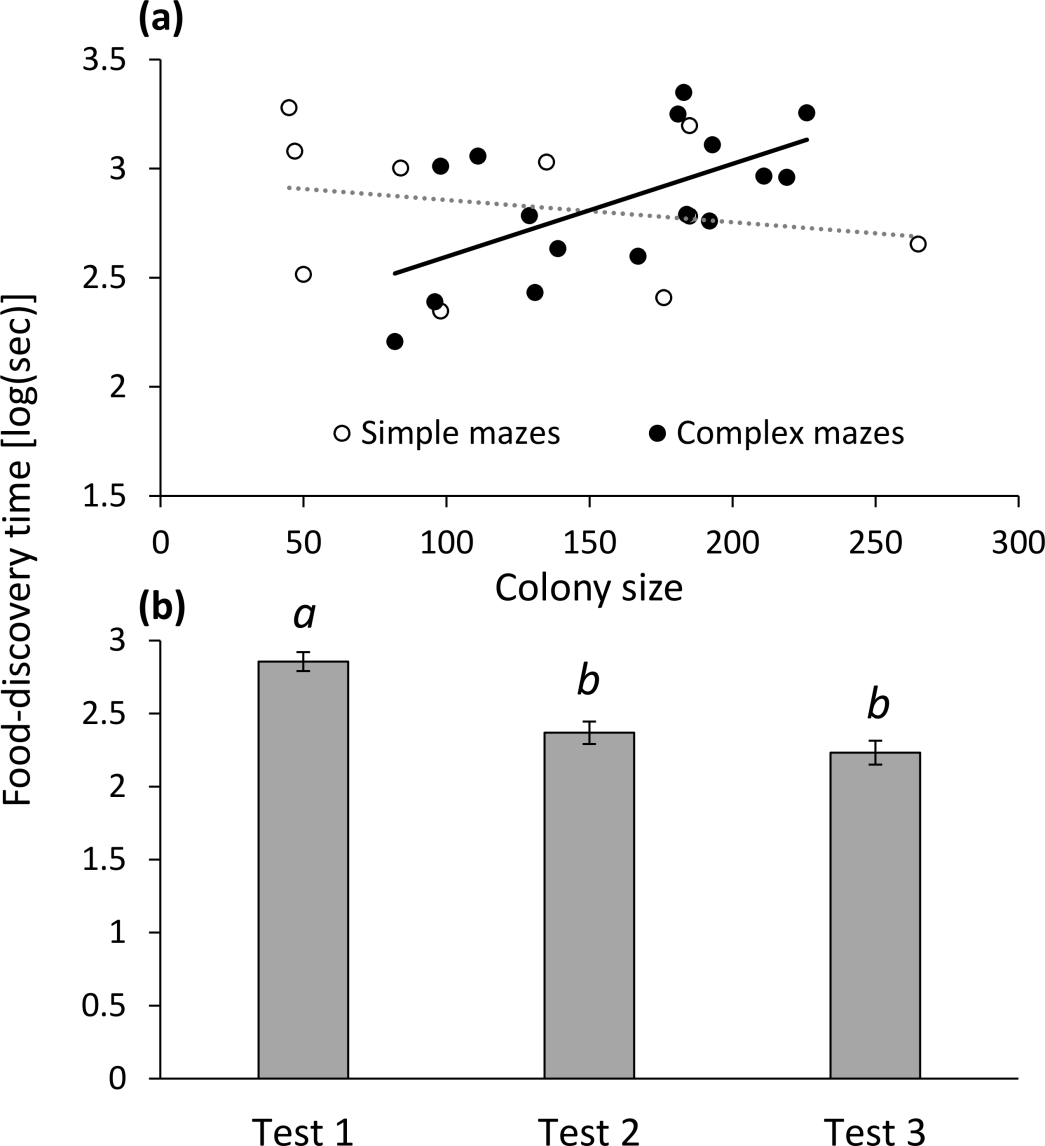
(a) The interaction between colony size and maze complexity in their effect on food-discovery time on the first test; (b) the decrease in food-discovery time with successive tests performed on the same day. Letters denote significant differences according to a post-hoc comparison.

Maze complexity interacted with colony size to affect food-discovery time: larger colonies took longer to solve the maze and reach the food, but only when searching in complex mazes (Fig 2A).

#### Workers searching & food consumption

Neither complexity level nor colony size had an effect on the number of workers searching or food consumption.

### Within-day changes in foraging response variables

Food-discovery time decreased within the first day of tests, indicated by the negative slope of the regression of food-discovery time on test number (mean: -0.3118, CI: [-0.4174, -0.2063], t = -6.097, df = 24, P < 0.001; Fig 2B). This indicates that colonies reached the food reward faster with recurring tests on the same day. The two other foraging response variables did not change within a single day (workers searching: mean: -0.0509, CI: [-0.1392, 0.0374], t = -1.189, df = 24, P = 0.246; food consumption: mean: 0.0281, CI: [-0.1727, 0.2289]), t = 0.292, df = 20, P = 0.774. Regarding the effect of colony size and maze complexity on the three foraging response variables, neither explanatory variable was ever significant (P > 0.218 for all explanatory variables; see ‘Supporting Information‘).

### Changes between successive days in foraging response variables

Statistical results are summarized in Table 2.

**Table 2:** The effect of colony size, maze complexity and day (1 vs. 2) on food-discovery time, the number of workers searching, and food consumption (N = 19 for food-discovery time and workers searching and 17 for food consumption).

	Food-discovery time	Workers searching	Food consumption
Between subjects			
Colony size	F_1,15_ = 0.089, P = 0.770	F_1,15_ = 16.856, **P < 0.001**	F_1,13_ = 0.441, P = 0.518
Maze complexity	F_1,15_ = 5.504, **P = 0.033**	F_1,15_ = 0.282, P = 0.603	F_1,13_ = 1.770, P = 0.206
Colony size × Maze complexity	F_1,15_ = 5.509, **P = 0.033**	F_1,15_ = 0.002, P = 0.963	F_1,13_ = 0.568, P = 0.464
Within subjects			
Day	F_1,15_ = 8.444, **P = 0.011**	F_1,15_ = 0.377, P = 0.548	F_1,13_ = 3.253, P = 0.095
Day × Colony size	F_1,15_ = 1.566, P = 0.230	F_1,15_ = 0.095, P = 0.762	F_1,13_ = 0.845, P = 0.375
Day × Maze complexity	F_1,15_ = 3.612, P = 0.077	F_1,15_ = 0.008, P = 0.932	F_1,13_ = 0.374, P = 0.551
Day × Colony size × Maze complexity	F_1,15_ = 2.563, P = 0.130	F_1,15_ = 0.045, P = 0.825	F_1,13_ = 0.330, P = 0.575

#### Food-discovery time

**Fig 3:**
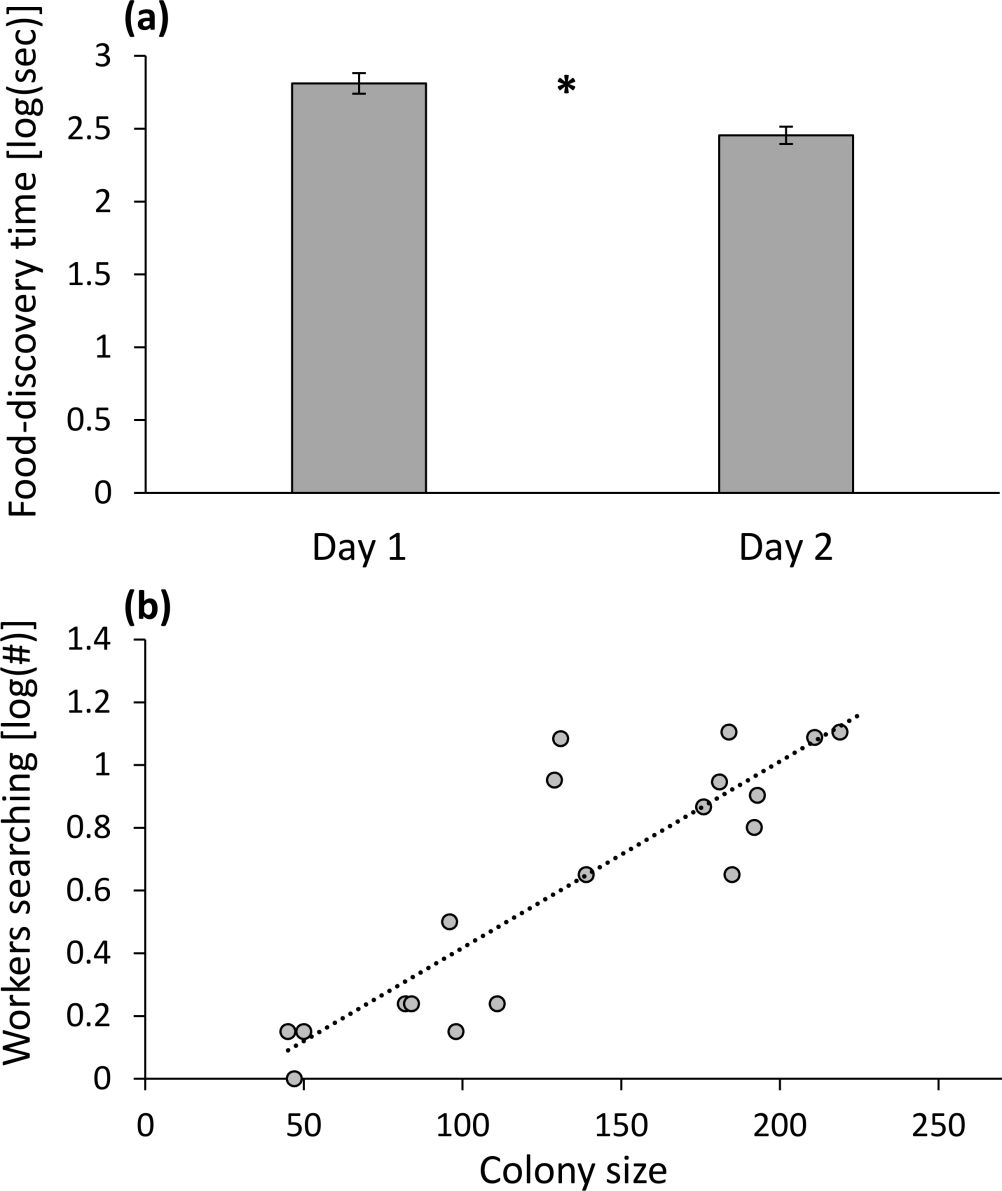
(a) The decrease in food-discovery time between the first tests on two successive days; (b) the positive correlation between colony size and the number of workers searching (mean values for the first tests on the two successive days are presented). The asterisk indicates a significant difference.

On the second day, mazes were solved faster and the food reward was reached faster compared to the first day (Fig 3A). Similar to the first analysis, the interaction term of colony size and maze complexity was significant (similar to Fig 2A).

#### Workers searching

The number of workers searching increased with colony size (Fig 3B).

#### Food consumption

Neither colony size, nor complexity or day had an effect on food consumption.

### Foraging response variables over longer time intervals

Statistical results are summarized in Table 3.

**Table 3:** The effect of day (before and after the time interval) and the time interval between days on the three foraging response variables. N = sample size (number of colonies).

Foraging variable (N)	Day	Time interval	Day × Time interval
Food-discovery time (18)	F_1,16_ = 10.087, **P = 0.006**	F_1,16_ = 0.727, P = 0.407	F_1,16_ = 5.400, **P = 0.034**
Workers searching (18)	F_1,16_ = 3.763, P = 0.070	F_1,16_ = 0.077, P = 0.785	F_1,16_ = 0.905, P = 0.356
Food consumption (17)	F_1,15_ = 0.268, P = 0.613	F_1,15_ = 0.003, P = 0.956	F_1,15_ = 1.169, P = 0.297

**Fig 4:**
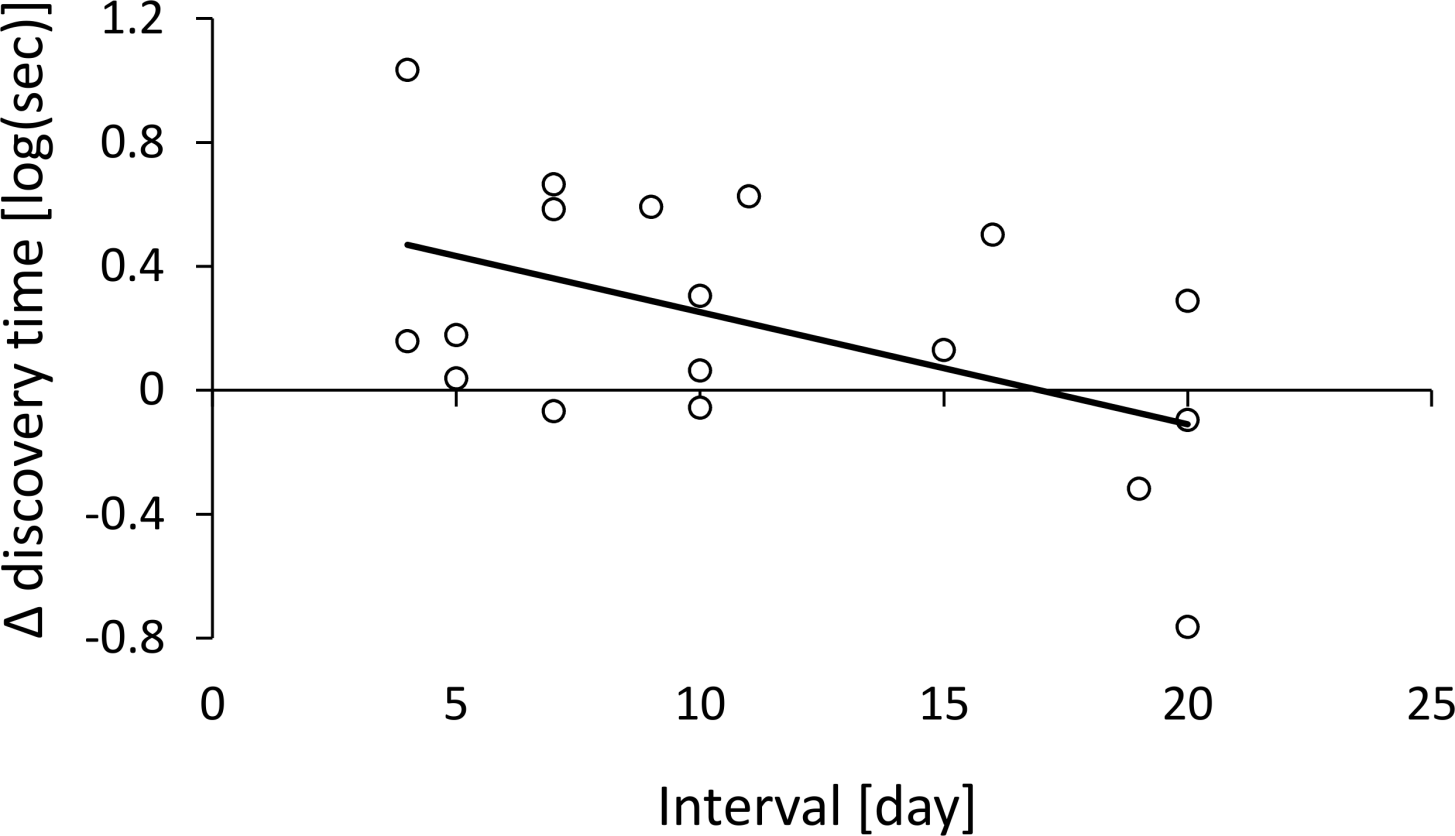
The effect of the time interval (in days, horizontal axis) between tests on the difference between food-discovery time before and after this interval (Δ discovery time, vertical axis). Values above zero indicate that colonies reached the food reward faster after the time interval than before it, while values below zero indicate that colonies reached the food reward faster before the time interval. As intervals become large, food-discovery time returns to the basic initial levels (around zero), which takes place according to this model after ~16 days.

Food-discovery time was longer for the first test on day 1 than for the first test following the longer time interval (4–20 days). However, the difference between the tests depended on the length of the time interval (the two-way interaction between time interval and test was significant): the shorter the interval, the stronger the decrease in food-discovery time between the first test on day 1 and the first test following the time interval (Fig 4). The two other foraging response variables did not differ between the two tests and were not affected by the time interval.

## Discussion

We demonstrate here the learning ability of *Cataglyphis niger* workers in the context of foraging within and between days. Learning ability was reflected in faster solving of the maze and arrival at the food reward, which can provide advantage under competition conditions. We show that the foraging workers do not only learn but also forget: while shorter time intervals between tests led to faster arrival at the food, longer intervals did not. Maze complexity had a relatively small effect on the results: maze complexity interacted with colony size to affect food-discovery time. Specifically, while colony size had little effect on food-discovery time when searching in simple mazes, larger colonies actually did worse than smaller ones under complex mazes.

In our experiments, not all measured foraging response variables equally changed with experience, but the pattern of change or no change was similar when compared within- and between-days. Food-discovery time decreased with successive tests on the same day and between days, while the number of workers searching or food consumption did not. The explanation we suggest for the contrast between decreasing food-discovery time and unchanging food consumption is that the immediate food reward was not the only motivation to solve the maze, but rather acquiring knowledge on the colony surroundings. In other words, the motivation for solving the maze could have been that of tracking the environment in order to locate new, temporary food resources, and also perhaps to become aware of any potential danger, rather than or in addition to discovering the honey offered. The lack of increase in food exploitation despite the faster food detection could be also explained by a small number of workers learning, no recruitment, and a quite short time allowed for food exploitation. Finally, it could be that colonies were more protein-deprived than sugar/carbohydrate-deprived and were therefore not interested in collecting honey. An interesting future direction would be to repeat this experiment with small dead insects as a reward. That said, animals that are protein-deprived sometimes compensate with overconsumption of carbohydrate-rich food in order to extract sufficient protein [42], which did not hold true here. The number of workers searching was strongly and positively correlated with colony size. This result may help estimating *Cataglyphis* colony size in the field, and though this result is not surprising, colony size is rarely taken into account in similar studies.

Memory duration is an important aspect of learning, and it takes place due to the cost of memory retention and the decrease of relevant information with time [14, 31]. Many other behaviors, such as color preference while foraging, choosing a suitable host for a parasitoid species, and elevated aggression towards a potential danger, fade with time [43–45]. It took the foraging workers in our set-up a little more than two weeks to forget how to solve the maze and return to the initial maze-solving times. It is interesting to compare this memory duration with either the rate of spatial change in resource locations, or the typical lifespan of *Cataglyphis* foragers in nature (only 10% still alive after two weeks in a congeneric species; [46]). That said, since we did not feed the colonies throughout the experiment, their starvation level increased with increasing time interval between the second day of the experiment and further tests. Generally, mild starvation leads to more intense foraging, while long starvation impairs foraging due to exhaustion [47]. We believe that starvation did not play an important role here, because if starvation took place, colonies that experienced a longer time interval between the second and next day of experiment (the interval was 4–20 days) should have collected more honey than colonies that experienced a shorter interval. This did not hold true. Colonies might have experienced mild starvation, but it should have increased the foraging effort and motivation, in contrast to our results, supporting the interpretation that the foraging workers simply forgot how to solve the maze. Future studies should test more thoroughly for the effect of starvation on learning and of starvation on the positive correlation between colony size and the number of workers searching. We expect a hump-shaped pattern of an increase followed by a decrease.

The interaction between maze complexity and colony size is intriguing: larger colonies took longer to solve the maze and reach the food, but only in complex mazes. Each time two workers encountered one another in the experiment while searching, they spent one-two seconds antennating (pers. obs.). Movement in complex mazes involved movement through narrower routes and larger colonies may have had more workers searching, perhaps leading to a longer time needed to solve the maze and discover the food on the first test. We therefore speculate that there is an intermediate number of foragers that will lead to the most efficient solving of our mazes. Too few foragers would be less likely, only by chance, to solve the maze than an intermediate forager group size. Too many foragers could suffer, as explained, from too many interactions and would have hard time moving through the narrow passages. This suggestion is only a speculation, and it remains to be tested by documenting such encounters and testing to which extent they slow down searching workers.

Colony size had little effect on foraging in our experiment, except for its positive correlation with the number of workers searching. Colony size is a key trait, correlated with foraging strategy, from individual foraging to trunk trail foraging, foraging intensity and distance, and level of specialization, to name just a few examples [48–50]. Smaller colonies can nevertheless compensate for their reduced work force by being more efficient. For example, per-worker productivity is higher in smaller than in larger colonies, and smaller colonies are more efficient during nest relocation [51–52]. These contrasting effects of colony size suggest that its effect is not straightforward but species-specific, and this may have been the cause of the little effect found here. Colony size might be more influential when searching over larger distances under more natural conditions. We believe that our framework of mazes increasing in complexity levels can be applied to different insects and be adapted to ask further questions, such as the contribution of learning when competing against naïve individuals or colonies.

## Supporting information

S1 Fig A simple mathematical description of the maze used in the experiment. (DOCX)

S1 Table The effect of colony size and maze complexity on the slope of the three foraging response variables on day 1. (DOCX)

S1 Data Data are avaiable as an excell file. (XLSX)
