# Supplementary material for: The interplay between maze complexity, colony size, learning and memory in ants while solving a maze: A test at the colony level
Source: PLoS One. 2017 Aug 24;12(8):e0183753. doi: 10.1371/journal.pone.0183753 (PMC5570381; doi:10.1371/journal.pone.0183753)
Supplement: S1 Fig — (DOCX) [file pone.0183753.s001.docx]

Supporting Information for the Manuscript (File 1):

**The interplay between maze complexity, colony size, learning and memory in ants while solving a maze: a test at the colony level**

By: Maya Saar, Tomer Gilad, Tal Kilon-Kallner, Adar Rosenfeld, Aziz Subach & Inon Scharf

The supporting information (File 1) contains:

A simple mathematical description of the maze used in the experiment (S1 Fig).

**(1) A simple mathematical description of the maze used in the experiment:**

The maze was constructed as a binary tree according to the following equation, when *D* is the sum of all decisions in the maze and *k* is the complexity level:

$$D=\sum{2_{1}^{0}+2}_{2}^{1}+2_{3}^{2}+2_{4}^{3}+\ldots+2_{n}^{k}$$

The general equation is:

$$D=\sum_{i=1}^{n} 2_{n}^{k}$$

The total number of correct decisions for each complexity level includes the first two decisions made: (I) to leave the nest, and (II) to enter the maze, and is:

*T* = *k* + 1

Let us take, for example, a maze of a complexity level of *k* = 3 (S1 Fig). The nest is placed at the left side, while the food reward is at the right side. The entry to the maze is marked with "I".


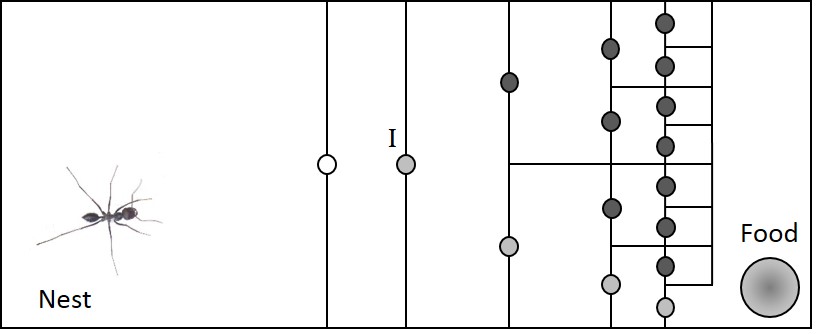


**S1 Fig**

The total number of decisions is *D* = 2^0^ +2^1^ +2^2^ +2^3^ = 15. The number of correct decisions is *T =* 1 + 3 = 4 (light grey circles). All other decisions (*D – T* = 15 – 4 = 11; dark grey circles) are wrong. The number of wrong decisions increases with *T*, but at an accelerating rate. This implies that as the complexity of the maze climbs, the time waste climbs up too, as the ratio of correct to wrong decisions decreases.
