# Supplementary material for: The interplay between maze complexity, colony size, learning and memory in ants while solving a maze: A test at the colony level
Source: PLoS One. 2017 Aug 24;12(8):e0183753. doi: 10.1371/journal.pone.0183753 (PMC5570381; doi:10.1371/journal.pone.0183753)
Supplement: S1 Table — (DOCX) [file pone.0183753.s002.docx]

Supporting Information for the Manuscript (File 2):

**The interplay between maze complexity, colony size, learning and memory in ants while solving a maze: a test at the colony level**

By: Maya Saar, Tomer Gilad, Tal Kilon-Kallner, Adar Rosenfeld, Aziz Subach & Inon Scharf

The supporting information (File 2) contains:

The effect of colony size and maze complexity on the slope of the three foraging response variables on the day 1 (S1 Table).

**S1 Table:**

The effect of colony size and maze complexity on the slope of the three foraging response variables on day 1 (three tests; corresponds to "within-day changes in foraging response variables" in the Results).

| Foraging variable | Colony size | Maze complexity | Interaction term | Model |
| --- | --- | --- | --- | --- |
| Food-discovery time | t = 0.236,  P = 0.816 | t = 0.496,  P = 0.625 | t = -0.534,  P = 0.599 | N = 25,  R^2^ = 0.014 |
| Workers searching | t = -0.657,  P = 0.518 | t = 1.271,  P = 0.218 | t = -0.726,  P = 0.476 | N = 25,  R^2^ = 0.152 |
| Food consumption | t = -0.245,  P = 0.810 | t = -0.860,  P = 0.402 | t = 1.040,  P = 0.313 | N = 21,  R^2^ = 0.079 |
